# EMOTIONAL RESPONSES OF ADULTS LIVING WITH DEMENTIA TO AN INTERGENERATIONAL MUSIC INTERVENTION

**DOI:** 10.1093/geroni/igad104.2408

**Published:** 2023-12-21

**Authors:** Jennie Dorris, Ketki Raina, Stephen Neely, Juleen Rodakowski

**Affiliations:** University of Pittsburgh, Pittsburgh, Pennsylvania, United States; University of Pittsburgh, Pittsburgh, Pennsylvania, United States; Carnegie Mellon University, Pittsburgh, Pennsylvania, United States; University of Pittsburgh, Pittsburgh, Pennsylvania, United States

## Abstract

Music interventions have shown promise to support emotional well-being in older adults living with dementia. Bolstering emotional well-being could improve the quality of life of the millions of older adults living with dementia. We implemented an intergenerational music intervention at Dementia360, a community program that serves older adults living with dementia. The music intervention featured adolescent musicians performing the preferred music of the older participants and engaging them in interactive musical activities via Zoom. 14 older adult participants attended an average of four sessions, 54 minutes each, of the intervention. We described their positive affect and pleasure as they engaged in the intervention. We compared participants’ scores on the Positive and Negative Affect Schedule (PANAS) pre- and post-intervention. We also assessed the participants’ emotions by rating them using the Observed Emotion Rating Scale (OERS). On average, the participants’ scores increased three points on the Positive Affect subscale of the PANAS. Our computed cohen’s d effect size was 0.5, a moderate effect size that correlates with self-report measures of feeling “a little more” to “much more” positive affect. On the OERS Pleasure subscale, we found that pleasure was shown for more than one minute of the ten-minute sample in over 87% of the observations. Intergenerational music interventions employ the safe, pleasurable, and scalable modality of music. These findings suggest that participation in such an intervention has the potential to influence positive affect and pleasure, critical supports for the emotional well-being of older adults living with dementia.

